# How Robust Is the Optimistic Update Bias for Estimating Self-Risk and Population Base Rates?

**DOI:** 10.1371/journal.pone.0098848

**Published:** 2014-06-10

**Authors:** Neil Garrett, Tali Sharot

**Affiliations:** Affective Brain Lab, Experimental Psychology, University College London, London, United Kingdom; Inserm, FRANCE

## Abstract

Humans hold unrealistically optimistic predictions of what their future holds. These predictions are generated and maintained as people update their beliefs more readily when receiving information that calls for adjustment in an optimistic direction relative to information that calls for adjustment in a pessimistic direction. Thus far this update bias has been shown when people make estimations regarding the self. Here, we examine whether asymmetric belief updating also exists when making estimations regarding population base rates. We reveal that while participants update beliefs regarding risk in the population in an asymmetric manner, such valence-dependent updating of base rates can be accounted for by priors. In contrast, we show that optimistic updating regarding the self is a robust phenomenon, which holds even under different empirical definitions of desirable information.

## Introduction

Humans are optimistically biased when making predictions about their future [1]–[8], including when estimating financial profits [1], relationship outcomes [2], longevity [3], professional success [4] and physical health [5]. In particular people habitually underestimate the likelihood of negative events in their lives and overestimate the likelihood of positive events (for review see [6]). This well-known bias, termed unrealistic optimism [7] is observed across age [8], culture [9], and species [10] and has a significant societal impact on domains ranging from financial markets [11], [12] to health and well-being [5].

Recently, we have proposed a mechanism by which unrealistic optimism arises and is persevered when confronted with counter-evidence [13]–[17]. Specifically, we have shown that people update their beliefs in an asymmetric manner – adjusting estimates more in response to desirable information about the future than undesirable (also shown by others, see [18], [19]). Over time such a mechanism will lead to positively skewed beliefs. The same mechanism has been demonstrated to underlie the “superiority illusion” [20]–[21] – the tendency to overestimate one's abilities and characteristics [22]. For example, Eil and Rao [21] showed that people adjust their beliefs regarding their level of intelligence and physical attractiveness when they receive information indicating they are more intelligent and attractive than they had assumed. However, they relatively fail to do so in response to information suggesting they rate lower on these attributes than they had previously thought [21].

An open question is whether the update bias exists only when adjusting beliefs about the self [13]–[16], [19], or whether it is observed also when adjusting beliefs about the population at large (base rates). This is important for understanding biases in risk estimation for two reasons. First, when estimating own risk people may incorporate both base rates and diagnostic information in their calculations. For example, if someone is estimating their likelihood of cancer they may consider the known frequency in their population together with knowledge about themselves (i.e. do I smoke? do I exercise?). Thus, a bias in updating base rates may effect updating for self risk. Second, it has been suggested that people tend to be optimistically biased when considering the self, less so when considering others [7]. It is thus of interest to examine whether or not the optimistic updating bias previously found for self risk will expand to base rates.

**Figure 1:**
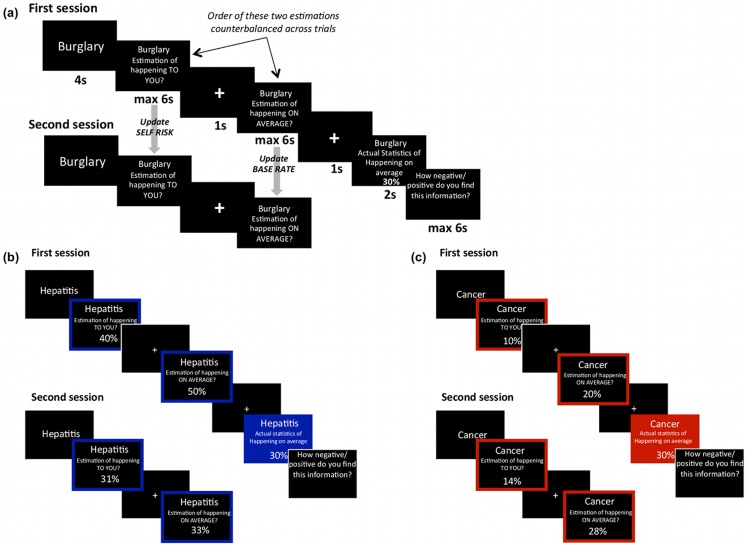
Paradigm. (a) In each trial, participants were presented with a short description of 1 of 80 adverse events and asked to estimate how likely this event was to occur **to** **themselves** in the future and how likely the event was to happen **on average** in the population. They were then presented with the base rate in a demographically similar population. Finally, participants were asked to rate how negative/positive they found this information. The second session was the same as the first except that the base rate was not presented and participants did not submit any ratings. Examples of trials in which the participant's estimate of the event occurring to themselves and the base rate was (b) higher or (c) lower than the provided base rate. In the specific examples shown here, under either classification scheme therefore these trials would be categorized as desirable and undesirable trials respectively.

To examine selective updating in estimating risk about oneself and the population we adjusted the belief update paradigm [16]. Participants completed a revised version of the belief update task where they estimated their own risk for 80 different negative events and also estimated the base rates of these events. On each trial they were then given explicit information regarding the base rates, and in a second session they estimated both again (see **Figure 1a** **, procedure**). The rationale in examining how participants update their estimates of base rates when receiving this information from the experimenter is that although a participant may recall the base rate presented accurately s/he may be uncertain of the validity of that information. For example, they may believe they have additional/more-up-to-date information regarding base rates that the experimenter does not know about. Thus when the participant incorporates the new information into his/her existing beliefs they may still do so in a biased manner.

**Figure 2:**
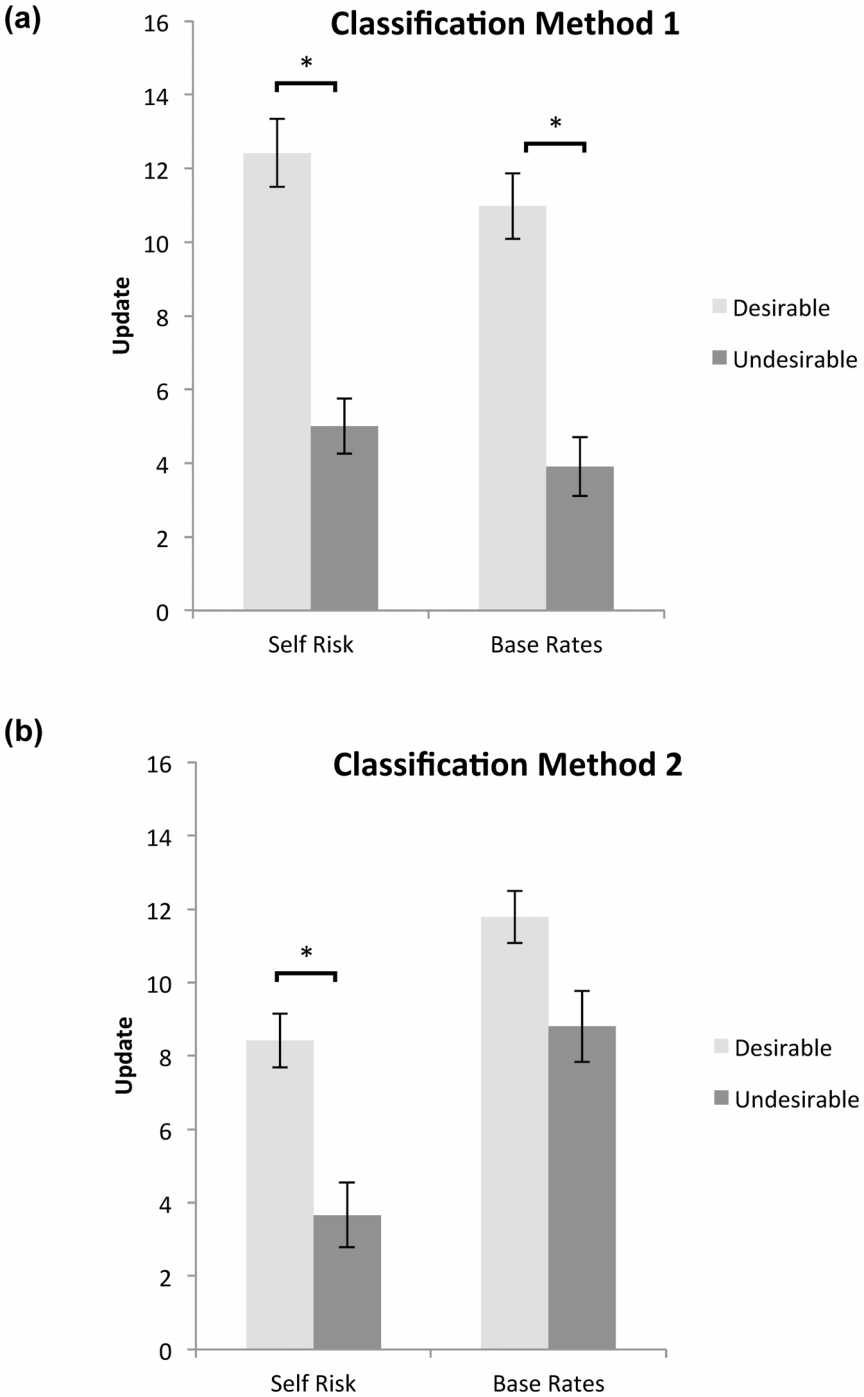
Update for self risk and base rates under different classifications of desirability. Participants update estimates of their self risk more when the information they received was desirable compared to undesirable. Participants update estimates of their base rates more when the information they received was desirable compared to undesirable under classification one but not under classification two after controlling for relevant covariates. Update calculated as first minus second estimation for desirable trials and the reverse for undesirable trials (positive values therefore indicate a move towards the information presented). Error bars are SEM; *indicates statistical significance at a threshold of P<0.05, two tailed after controlling for all relevant covariates. Trials classified as desirable when the participant overestimated the probability of the event occurring and undesirable when the participant underestimated the probability of the event occurring: (a) *to themselves* relative to the provided base rate; (b) *in the population* relative to the provided base rate.

The paradigm enabled us to quantify how participants adjust their beliefs about the self and the population in response to new information in two instances; (1) when they learn that the average likelihood of encountering a negative life event is lower than their own estimates (desirable news, **Figure 1b**) and (2) when it is greater (undesirable news, **Figure 1c**). To examine the robustness of the bias we further investigated if the results differ if valence is empirically defined in two different ways: (1) by comparing the information presented, to the participants' estimate of their own probability of encountering a negative event (2) by comparing the information presented to the participants' estimate of the population base rate. By asking participants to rate the extent to which they found the information presented to them desirable or undesirable we could also examine whether these subjective ratings are driven more or less by deviations from: (1) estimations of self risk (2) estimations of the population base rate. Finally, additional experimental factors that may influence the results (such as memory for the information provided and priors) were tested.

## Materials and Methods

### Participants

The study was approved by the UCL Psychology Ethics Committee. Written informed consent was obtained from all participants. Thirty two individuals aged 18 to 33 participated in the study (mean age  = 22.93; sd  = 3.64). An additional six participants originally completed the task but were excluded due to Beck Depression Scores above 12, indicating possible major depression disorder. This is due to previous findings showing a lack of update bias in depressed individuals [23]. All participants were recruited from UCL psychology subject pool. Participants were paid for their participation.

### Stimuli

Stimuli consisted of eighty short descriptions of adverse life events (e.g. passenger in a car accident, home burglary – see [16]). For each adverse event, the average probability of that event occurring at least once to a person living in the same socio-cultural environment as the participants was determined from online resources (Office for National Statistics, Eurostat, PubMed). Very rare, or very common, events were not included; all events probabilities lay between 10% and 70%. To ensure that the range of possible overestimation was equal to the range of possible underestimation, participants were told that the range of probabilities lay between 3% and 77%.

### Procedure

The paradigm was modified from our previous studies [13]–[16] and depicted in **Figure 1**. Participants completed a practice session before beginning the main experiment. On each trial, one of 80 stimuli was presented on screen for 4s. Participants were then asked to separately estimate how likely the event was to happen to **themselves** in the future and how likely the event was to happen **on average** in the population. In half of the trials the order of these estimations was reversed (i.e. participants were first asked to estimate how likely the event was to happen on average and then to estimate their own likelihood). Participants were then shown the actuarial frequency of the event in a demographically similar population for 2s. Finally, participants were asked to rate on a 7 point scale (1 = Very negative; 7 =  Very Positive) how negative/positive they found this information. Participants had up to 6s to give each estimation and rating. If the participant failed to submit a response for either estimation or rating, that trial was excluded from all consequent analyses (mean trials with missing response  = 2.50, s.d.  = 2.78).

In a second session, immediately after the first, participants were asked again to provide estimates of their likelihood and the average likelihood of encountering the same events (order reversed in half the trials) so that we could assess how they updated both estimations regarding the self and estimations regarding base rates.

In half of the trials, participants estimated the likelihood of the event *happening* to them and on average in the future. In the other half of trials, participants estimated the likelihood of the event *not happening* to them and on average in the future. We framed estimations in these two ways so that differential updating could not be attributed to differential processing of high and low numbers. Furthermore, under such framing half the trials were conceptually presented as negative events (i.e. divorce) and half as positive events (i.e. – never divorce).

After completing the task, participants rated all stimuli on prior experience [for the question “Has this event happened to you before?” the responses ranged from 1 (never) to 6 (very often)], familiarity [for the question “Regardless if this event has happened to you before, how familiar do you feel it is to you from TV, friends, movies, and so on?” the responses ranged from 1 (not at all familiar) to 6 (very familiar)] and negativity [for the question “How negative would this event be for you?” the responses ranged from 1 (not negative at all) to 6 (very negative)]. To test memory for the information presented, participants were asked to provide the actual probability previously presented of each event.

### Data Analysis

All statistical percentages and all responses in the ‘not happen’ sessions were transformed into the corresponding numbers of the ‘happen’ sessions by subtracting the respective number from 100. Trials were divided into ones in which participants received desirable or undesirable information using 2 different classification criteria:

1. Trials were classified as desirable when the participant initially overestimated the probability of the event occurring to *themselves* relative to the provided base rate. Conversely, trials were classified as undesirable when the participant initially underestimated the probability of the event occurring to themselves relative to the provided base rate. Trials in which participants' estimates of their own likelihood were exactly equal to the provided base rate were excluded from the analysis (mean number excluded trials  = 1.72; s.d.  = 1.25).
2. Trials were classified as desirable when the participant initially overestimated the base rate relative to the provided base rate. Trials were classified as undesirable when the participant underestimated the base rate relative to the provided base rate. Trials in which participants' estimates of the base rate were exactly equal to the provided base rate were excluded from the analysis (mean number excluded trials  = 1.53; s.d.  = 1.02).

For both of these classifications, we calculated update terms for desirable trials as: 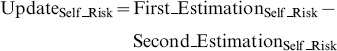


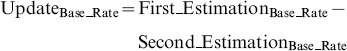



For undesirable trials, update terms were calculated as:
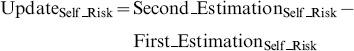


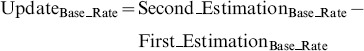



Update scores were entered into ANOVAs and follow up t-tests were conducted.

Memory errors were calculated as the absolute difference between the probability previously presented and the participants' recollection of that statistic:
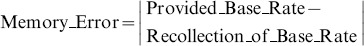



Finally we calculated two estimation error terms quantifying for each trial the difference between participants' initial estimates and the information presented:
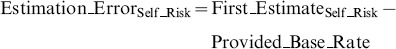


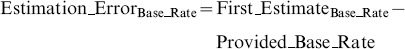



To test whether desirability ratings were driven more or less by the extent to which the statistical information differed from participants' estimations regarding the self or regarding base rates, for each participant we separately correlated each set of estimation errors with their desirability ratings. To statistically test for a difference in the strength of these correlations accounting for the additional correlation between the 2 sets of estimation errors we compared these using Steiger's Z-test.

## Results

### i. Is updating beliefs regarding personal risk & base rates biased?

As detailed below, our results show biased updating for self risk **(**see **Figure 2** **)**. Specifically, updating for self risk is greater in response to desirable information relative to undesirable information under both classification schemes of desirability. However, for base rates biased updating can be accounted for by priors.

#### (a) Updating of personal risk

Replicating previous findings [13]–[16] classification scheme 1 revealed that participants updated their beliefs regarding self risk more when the information regarding base rates was better than their estimate of self risk compared to when it was worse (t(31)  = 6.09, P<0.01). This bias persists under classification 2 when classifying desirability of information as dependent on base rates (i.e. if information regarding base rates is greater or smaller than participants' estimate of these base rates). Participants were more likely to update their beliefs about their own likelihood of encountering a negative life event when the base rate was better than their initial estimate of the base rate compared to trials in which the base rate was worse (t(31)  = 4.83, P<0.01). These results replicate our previous findings of valence dependent updating [13]–[16] and confirm that valence dependent updating for self risk is not contingent on classification scheme.

As detailed below, these findings hold after accounting for possible confounding factors. Specifically, as we describe in section C, examining all additional factors and ratings revealed four possible confounds: (1) under classification 1 ratings of past experience differed for stimuli for which subjects received desirable and undesirable information; (2) under classification 2 magnitude of estimation errors differed for stimuli for which subjects received desirable and undesirable information; (3) under classification 2 there were a greater number of trials for which subjects received desirable information than undesirable information (4) initial estimates of personal risk and base rates differed (this is true for both classifications). When controlling for these additional factors in the respective classifications a main effect of valence remained (classification 1: F(29)  = 8.02, P<0.01, classification 2: F(29)  = 10.72, P<0.01) confirming the robustness of the bias.

#### (b) Updating of base rates

Participants updated their beliefs about the base rate more when the presented base rate was better than their estimate of self risk, compared to trials in which the base rate was worse (i.e. classification 1: t(31)  = 5.58, P<0.01) and also when the actual base rate was better than their initial estimate of this base rate compared to when it was worse (i.e. classification 2: t(31)  = 2.43, P<0.03). As detailed below, however, the finding is abolished under classification two when accounting for priors.

Specifically, as we describe in section C, examining all additional factors and ratings revealed four possible confounds: (1) under classification 1 ratings of past experience differed for stimuli for which subjects received desirable and undesirable information; (2) under classification 1 and 2 magnitude of estimation errors differed for stimuli for which subjects received desirable and undesirable information; (3) under classification two the number of trials for which subjects initially overestimated the base rate and thus received desirable information was greater than the number of trials in which they underestimated it and thus received undesirable information; (4) initial estimates of personal risk and base rates differed (this is true for both classifications). When controlling for these additional factors in the respective classifications a main effect remained under classification 1 (F(29)  = 4.59, p<0.05), but not 2 (F(29)  = 0.64, p>0.4). Looking at the latter in detail revealed that biased updating of base rates was mostly contingent on the differences in the number of trials for which subjects received desirable information and undesirable information. With that covariant alone biased updating for base rates was abolished (F(29)  = 2.37, p>0.13). Without it the effect remained (F(29)  = 4.12 p = 0.05).

#### (c) Other variables (memory, familiarity, past experience, perceived negativity, estimation errors, number of trials)

Below we detail our examination of any experimental factors (i.e. memory, familiarity, past experience, perceived negativity, estimation errors, priors, number of trials) that might differ for trials in which subjects received desirable and undesirable information. We do this under both classification schemes and for both estimations of self risk and base rates. As we described in sections a + b, update bias was re-examined after controlling for any differences found in these variables.

Memory: To examine participants' memory of the information given, at the end of the session, participants were asked to indicate the actual probability (as previously presented) of each event occurring to an average person in the same socio-cultural environment. Memory errors were calculated as the absolute difference between the actual probability previously presented and participants' recollection of that statistical number. Participants remembered information presented to them equally well, irrespective of whether it was desirable or undesirable and irrespective of whether desirable and undesirable was classified according to method 1 (t(31)  = 0.68, P> 0.50) or method 2 (t(31)  = 0.01, P>0.99).

Note that a participant may recall the base rate presented accurately but be uncertain of the validity of that information. For example, they may believe they have additional/more-up-to-date information regarding base rates that the experimenter does not know about. Thus recollection of these numbers and the participant's second estimate of the base rate may differ. Comparing these two scores (i.e. recollection of base rates presented and second estimation of base rates) revealed they were not significantly different from each other, but there was a trend (t(31) = −1.76, p = 0.09).

Familiarity, perceived negativity, past experience: Questionnaire scores revealed that participants did not rate events for which they received desirable and undesirable information as differing in familiarity (i.e. how familiar they are with the stimuli from friends, family TV etc.) or negativity (how negative they perceive the event to be) under either classification method (see Table 1). However, under classification one participants rated events for which they received desirable information as greater on past experience compared to events in which they received undesirable information (t(31)  = 3.02, P<0.01). We controlled for this difference in sections a + b.

**Table 1:** Participants' ratings of familiarity, prior experience, negativity, memory errors, initial estimates, number of trials and estimation errors.

	Classification 1 mean (SD)		Classification 2 mean (SD)	
Questionnaire and variables	Desirable	Undesirable	Desirable	Undesirable
*Subjective Scales Questionnaire: All scales 1 = low to 6 = high*				
Familiarity	4.05 (1.04)	3.93 (1.16)	3.99 (1.09)	3.97 (1.16)
Prior experience	1.39 (0.37)*	1.23 (0.33)*	1.28 (0.31)	1.33 (0.36)
Negativity	3.97 (0.85)	4.09 (0.92)	4.05 (0.88)	4.01 (0.91)
*Task-related variables*				
Number of Trials	36.75 (13.60)	39.03 (12.66)	45.38 (8.90)*	30.59 (8.35)*
Memory errors	14.06 (5.89)	13.46 (3.81)	13.83 (5.91)	13.82 (4.10)
Initial estimate *self risk*	44.28 (6.83)*	19.30 (5.28)*	35.79 (9.03)*	24.47 (8.05)*
Initial estimate *base rates*	41.97 (5.80)*	29.40 (5.17)*	41.94 (5.02)*	26.19 (5.02)*
Estimation Error *self risk*	20.37 (4.79)	18.90 (2.63)	18.32 (4.54)*	21.73 (4.23)*
Estimation Error *base rates*	20.47 (3.83)*	15.43 (2.00)*	18.87 (3.79)*	16.68 (2.63)*

*significant difference between desirable and undesirable variable (p<0.05) within same classification.

Priors (first estimates and number of trials): In accordance with past research (e.g. see 7), participants believed their own likelihood of encountering a negative event was lower than their estimate of the base rate (initial estimate of self risk was lower than estimated base rate (t(31) = −4.30, P<0.01). This was observed in 84% of the participants, suggesting they believed they would fare better than average.

Furthermore, under classification 2 they would often overestimate the base rate relative to the base rate presented to them, such that the number of trials in which they received desirable information regarding base rates was larger than undesirable (i.e. ratio of desirable trials to all trials was larger than 0.5 t(31)  = 4.93, p<0.01). There were no significant differences in the number of trials in which they received desirable and undesirable information for self risk. We controlled for these differences in sections a + b.

Estimation Errors: As the magnitude of the update is likely to be related to the magnitude of the initial estimation error (i.e. the difference between the participant estimate and the information provided) it is critical to examine for differences in the magnitude of the errors for desirable and undesirable trials.

Under classification two there was a difference between desirable and undesirable estimation errors for self risk (t(31)  = −2.52, P<0.02). Under both classifications there were differences between desirable and undesirable estimation errors for base rates (classification one: t(31)  = 6.40, P<0.01; classification two: t(31)  = 2.40 p<0.03). We controlled for these difference in sections a + b.

### ii. Do estimation errors underlie desirability and update?

#### (a) Updating and Estimation Errors

Formal models suggest that learning from information that disconfirms one's expectations is mediated by a prediction error signal that quantifies a difference between expectation and outcome [24]–[26]. We have previously shown that an analogous mechanism underpins belief updating in this task [16]. Specifically, the difference between participants' initial estimations and the information provided (that is, estimation error) predicts subsequent updates, as would be expected from learning models [24]. The strength of this association is indicative of learning. We have shown that such learning is valence-dependent, being greater for information that offers an opportunity to adopt a more optimistic outlook than for information that calls for a more pessimistic outlook [16].

Here, we ask if updating for beliefs regarding the self and base rates are better predicted by estimation errors derived from beliefs regarding the self and base rates, and how this interacts with valence. To this end we conducted linear regressions for each participant with both Estimation Errors of Self Risk (unsigned) and Estimation Errors of Base Rate (unsigned) predicting: (1) Desirable Update of self risk, (2) Undesirable Update of self risk, (3) Desirable Update of base rate, (4) Undesirable Update of base rate, under both classifications. Regression coefficients were than tested at the group level.

**Figure 3:**
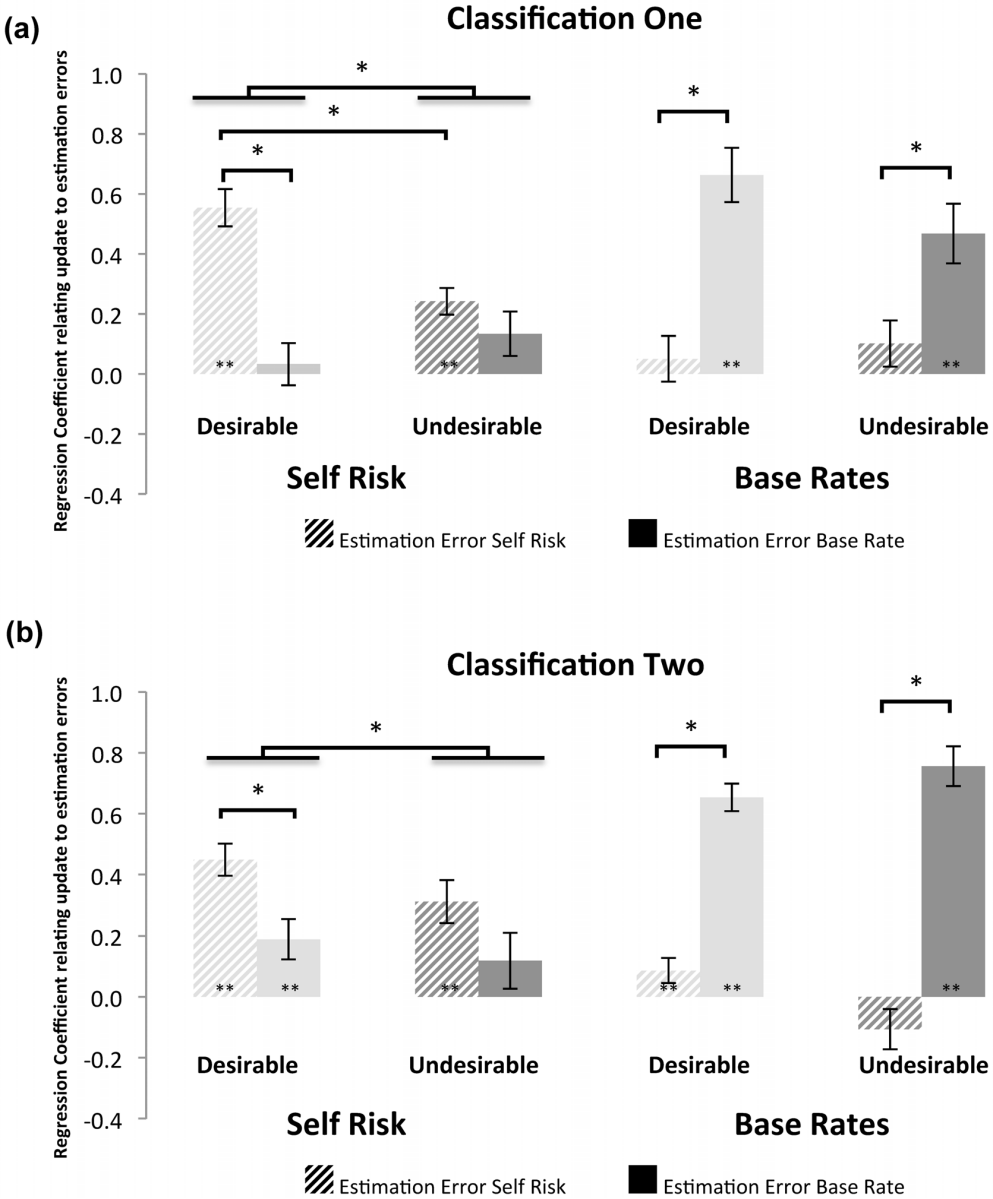
Regression coefficients predicting update from estimation errors. Estimation Error of *self* risk (i.e. the difference between a participants' estimate of self risk and provided base rate) significantly predicted update of self risk both for trials in which subjects received desirable and undesirable information, and under both classifications. Estimation Error of *base rate* (i.e. the difference between a participants' estimate of base rate and provided base rate) significantly predicted update of base rate both for trials in which participants received desirable and undesirable information, and under both classifications. Error bars are SEM; * indicates statistical significance at a threshold of P<0.05, two tailed paired sample t-test after controlling for relevant factors; ** indicates significantly different to a mean of 0, one sample t-test (P<0.05).

As seen in **Figure 3** estimation errors for self risk were better at predicting update for self risk and estimation errors for base rates were better at predicting update for base rates. In addition, the strength of the association between self-estimation errors and self-updating was valence dependent under classification one (t(31)  = 4.60, p<0.01), replicating previous findings [16]. Under classification 2 base rate estimation errors gain more predictive power in explaining some of the variance previously explained by self estimation errors. This resulted in neither type of estimation error alone showing a valence dependent difference in predicting self update. Rather, there was a main effect of valence such that estimation errors (of base rates and self together) were more predictive of update in response to desirable than undesirable information (F(31)  = 4.78, p<0.05).

#### (b) Desirability and estimation errors

Participants rated how desirable information was. We ask whether subjective desirability ratings were driven by the extent to which the statistical information differed from participants' estimates self risk, estimates of base rates, or both.

For each participant we separately correlated each set of estimation errors with their desirability ratings across trials. There was a positive correlation between desirability ratings and estimation errors for self risk (mean r  = 0.50, significantly different from zero across the population p<0.01) and estimation errors for base rate (mean r = 0.55, significantly different from zero across the population p<0.01). Specifically, participants rated information as increasingly desirable as the information provided diverged from their own estimate such that the former was a lower number. Steigers Z did not reveal a significant difference between the two sets of correlations (Z = −0.5, p>0.60), suggesting that desirability is associated with both.

### iii. Effects of Question Order and Frame

To examine whether the question order (i.e. if subject estimated their own likelihood first and then base rate or vice versa) and frame (i.e. if they were required to estimate likelihood of the event happening or not happening) influenced updating we conducted a 3 way repeat measure ANOVA on updating scores entering question order (self estimate/base rate first), frame (happen/not happen) and valence (desirable/undesirable) as repeated factors under each classification. Two effects were revealed.

(1) An interaction between valence and order for updating self risk under classification two (F(31) = 7.14, p<0.02) and a trend under classification one (F(31) = 4.03, p = 0.05) was observed. The interaction was characterized by greater valence-dependent updating when subjects estimated their own vulnerability before estimating base rate. This interesting result suggests that biased updating is reduced yet still significant when we first consider population base rates and only then our own likelihood (classification one; F(31) = 20.67, p<0.01; classification two; F(31) = 6.39, p<0.05). This may be because initial self-estimates tend to be more accurate when reported after estimates of base rates (t(31)  = 1.74, p = 0.09) and/or because undesirable information of base rates may be more difficult to ignore under such ordering. (2) A main effect of frame for updating of base rates was found under classification one (F(31) = 5.40, p<0.05) with updating for “happening” being greater than updating for “not happening”. However, the effect was not significant under classification two, nor for self risk under either classification.

## Discussion

Our results replicate past studies from our lab [13]–[16] and others [18]–[19] in showing that individuals selectively update their beliefs when estimating their own risk; updating their estimates more in response to information that offers an opportunity to adjust predictions in an optimistic direction relative to information that can reduce optimism. Importantly, our results further show that valence dependent updating of self risk reported previously [13]–[16] is not contingent on the specific method by which trials are divided into “desirable” and “undesirable”. In our original task [16] participants estimate their own probability of encountering negative events and then receive information regarding the population base rates. Trials are then labelled “desirable” if the base rates provided are better than the participants' estimate of self risk and “undesirable” if the base rates provided are worse than the participants' estimate of self risk. Such a division has proved useful when examining how the brain codes for the difference between ones' estimate of self risk and information regarding base rates. Specifically, we have found in the past that the left inferior frontal gyrus, medial frontal lobe and cerebellum track the magnitude of the difference between a person's estimate of self risk and information regarding base rates when that information is better than the person's estimate of their own vulnerability, while the right inferior frontal gyrus codes for the magnitude of such errors when the information is worse [16].

One can imagine a scenario where an individual holds a different estimate of their self risk and of the base rate and receives information regarding the base rate that is worse than their estimate of their self risk but better than their estimated base rate. Under this scenario whether the information is desirable or not may be ambiguous. In this study by asking participants to label the information themselves as desirable or undesirable and dividing the trials in two different ways (according to the participants' estimates of their self risk and according to the participants' estimates of base rates) we show that the two are highly correlated and that the update bias exists under both methods of division.

It has been suggested that when estimating vulnerability a person may take into account both population base rates and diagnostic information to reach a prediction regarding personal risk [27]–[28]. Thus, information regarding base rates will result in adjusting both estimates of personal risks and estimated base rates. Selective updating of either may lead to biased estimates for self risk. Here, we show that updating of beliefs regarding population base rates is not as robust and clear-cut as updating for self risk. Specifically, biased updating for base rates could partially be explained by priors; subjects tended to overestimate base rates such that there were more trials in which they ended up receiving desirable information. When accounting for this difference the bias for updating base rates did not survive under classification 2.

Ample evidence suggests that people's perception of their vulnerability is biased in a positive direction [30]. This study supports these past findings and demonstrates the robustness of the effect as it is observed under different empirical definitions of desirability of information provided. A bias in updating estimates of self risk can have adaptive benefits that include increasing explorative behaviour and reducing stress and anxiety, a factor that has links with physical and mental well-being [5], [29]. However, any advantage arising out of a reduced tendency to learn from undesirable information is likely to come at a cost. A pertinent example is the discounting of warning signs regarding financial risk, which is widely perceived as a contributing factor to the 2008 global economic collapse [31]–[32].
